# Supplementary material for: Saline gargle collection method is comparable to nasopharyngeal/oropharyngeal swabbing for the molecular detection and sequencing of SARS-CoV-2 in Botswana
Source: Microbiol Spectr. 2025 May 22;13(7):e02023-24. doi: 10.1128/spectrum.02023-24 (PMC12211010; doi:10.1128/spectrum.02023-24)
Supplement: Supplemental figure and tables — Figure S1 and Tables S1 to S3. [file spectrum.02023-24-s0001.docx]

**SUPPLEMENTARY MATERIAL**


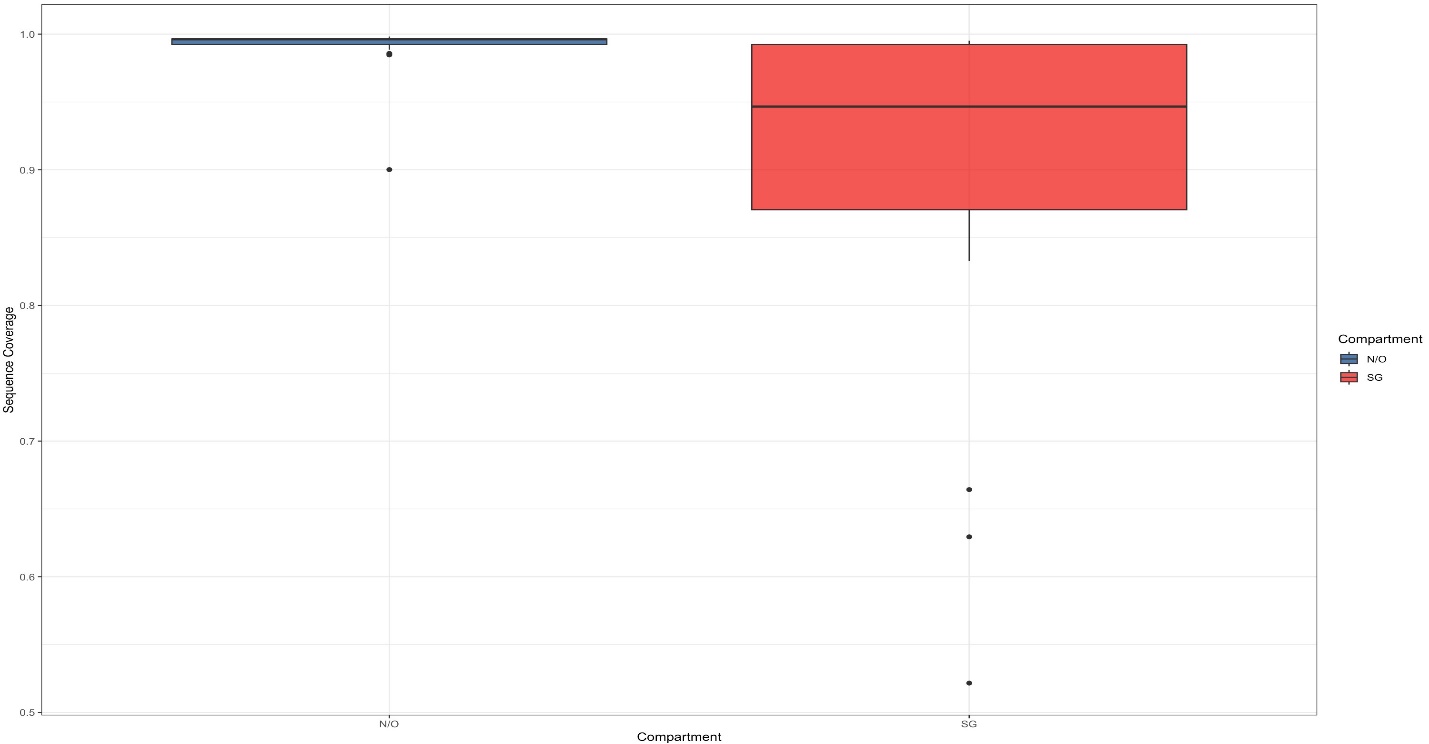


***Supp Figure 1****: Box plot of sequenced genome coverage comparison between SG and NOS collected samples. The SG specimen had a median genome coverage of 94.7% (IQR 87.0-99.2%), and the NOS specimen had a median genome coverage of 99.6% (IQR 90.0-99.6%). The SG specimen had 3 coverage outliers in samples KJL013 (62.9%), KJL027 (52.2%), and KJL33 (66.4%). The NOS specimen 2 coverage outliers in samples KJL11(90.0%) and KJL33 (98.5%).*

**Supp Table 1: Breakdown of all participant demographics**

| **Study ID** | **Age** | **Gender** | **Date of Recruitment** | **Reason for Test** | **Date of Onset** | **Testing Assay** | **Mean Ct (NOS)** | **Mean Ct (SG)** |
| --- | --- | --- | --- | --- | --- | --- | --- | --- |
| 001 | 5 | M | 24/12/2021 | symptomatic (cough) | 21/12/2021 | DaAN Gene | Negative | 41.7 (Negative) |
| 002 | 12 | F | 24/12/2021 | symptomatic (sore throat) | 21/12/2021 | DaAN Gene | 37.3 (Negative) | Negative |
| 003 | 5 | M | 24/12/2021 | symptomatic (blocked nose) | 22/12/2021 | DaAN Gene | Negative | 42.2 (Negative) |
| 004 | 16 | M | 24/12/2021 | contact tracing, no symptoms | n/a | DaAN Gene | Negative | Negative |
| 005 | 7 | F | 24/12/2021 | routine check, no symptoms | n/a | DaAN Gene | Negative | Negative |
| 006 | 13 | M | 24/12/2021 | routine check, no symptoms | n/a | DaAN Gene | Negative | Negative |
| 007 | 14 | F | 27/12/2021 | symptomatic (cough, headache, fever, sore throat) | 25/12/2021 | DaAN Gene | 16.5 (Positive) | 27.9 (Positive) |
| 008 | 14 | F | 27/12/2021 | symptomatic (cough, headache, fever) | 25/12/2021 | DaAN Gene | 17.6 (Positive) | 22.9 (Positive) |
| 009 | 6 | F | 28/12/2021 | travel purposes, no symptoms | n/a | DaAN Gene | Negative | Negative |
| 010 | 12 | M | 28/12/2021 | symptomatic (cough, headache) | 18/12/2021 | DaAN Gene | 30.6 (Positive) | Negative |
| 011 |  | M | 29/12/2021 | Second test | n/a | DaAN Gene | Negative | Negative |
| 012 | 9 | F | 29/12/2021 | Second test | n/a | DaAN Gene | Negative | Negative |
| 013 | 14 | F | 29/12/2021 | Second test | n/a | DaAN Gene | Negative | Negative |
| 014 | 7 | M | 29/12/2021 | Second test | n/a | DaAN Gene | Negative | Negative |
| 015 | 5 | F | 29/12/2021 | Second test | n/a | DaAN Gene | Negative | Negative |
| 016 | 11 | M | 29/12/2021 | symptomatic (cough, headache, running nose) | 25/12/2021 | DaAN Gene | 17.7 (Positive) | 30.2 (Positive) |
| 017 | 11 | M | 29/12/2021 | contact tracing, no symptoms | n/a | DaAN Gene | 31.5 (Positive) | 40.2 (Negative) |
| 018 | 8 | M | 29/12/2021 | contact tracing, no symptoms | n/a | DaAN Gene | Negative | Negative |
| 019 | 5 | F | 29/12/2021 | symptomatic (cough, fever) and contact tracing | 26/12/2021 | DaAN Gene | 17.8 (Positive) | 27.5 (Positive) |
| 020 | 14 | M | 29/12/2021 | Second test | n/a | DaAN Gene | Negative | Negative |
| 021 | 12 | F | 29/12/2021 | Second test | n/a | DaAN Gene | 19.3 (Positive) | 30.2 (Positive) |
| 022 |  | M | 29/12/2021 | Second test | n/a | DaAN Gene | 40.2 (Negative) | 41.2 (Negative) |
| 023 | 7 | M | 29/12/2021 | symptomatic (running nose) and contact tracing | 28/12/2021 | DaAN Gene | Negative | Negative |
| 024 | 7 | F | 29/12/2021 | contact tracing, no symptoms | n/a | DaAN Gene | Negative | Negative |
| 025 | 10 | F | 29/12/2021 | contact tracing, no symptoms | n/a | DaAN Gene | Negative | Negative |
| 026 | 16 | M | 29/12/2021 | symptomatic (headache, fever, running nose, sneezing) | 28/12/2021 | DaAN Gene | 21.7 (Positive) | 31.1 (Positive) |
| 027 | 16 | M | 29/12/2021 | contact tracing, no symptoms | n/a | DaAN Gene | Negative | Negative |
| 028 | 13 | M | 31/12/2021 | contact tracing, no symptoms | n/a | DaAN Gene | Negative | Negative |
| 029 | 10 | M | 31/12/2021 | contact tracing, no symptoms | n/a | DaAN Gene | Negative | Negative |
| 030 | 6 | M | 31/12/2021 | symptomatic (cough) | 27/12/2021 | DaAN Gene | Negative | Negative |
| 031 | 9 | F | 31/12/2021 | symptomatic (sore throat) | 27/12/2021 | DaAN Gene | Negative | Negative |
| 032 | 15 | F | 31/12/2021 | symptomatic (cough, sore throat) | 27/12/2021 | DaAN Gene | Negative | Negative |
| 033 | 11 | F | 31/12/2021 | symptomatic (cough) and contact tracing | 27/12/2021 | DaAN Gene | 21.9 (Positive) | 26.4 (Positive) |
| 034 | 14 | F | 31/12/2021 | symptomatic (cough, sore throat) and contact tracing | 29/12/2021 | DaAN Gene | 21.2 (Positive) | 32.0 (Positive) |
| 035 | 17 | M | 31/12/2021 | symptomatic (cough, fever) and contact tracing | 26/12/2021 | DaAN Gene | 21.7 (Positive) | 25.2 (Positive) |
| 036 |  | F | 31/12/2021 | symptomatic (cough, chest pains) | 29/12/2021 | DaAN Gene | 20.4 (Positive) | 27.1 (Positive) |
| 037 | 14 | F | 03/01/2022 | symptomatic | 31/12/2021 | DaAN Gene | Negative | Negative |
| 038 | 9 | M | 03/01/2022 | symptomatic | 02/01/2022 | DaAN Gene | Negative | Negative |
| S39 | 13 | M | 03/01/2022 | symptomatic | 31/12/2021 | DaAN Gene | Negative | Negative |
| 040 | 16 | M | 03/01/2022 | contact tracing, no symptoms | n/a | DaAN Gene | 25.5 (Positive) | 29.2 (Positive) |
| 041 | 14 | F | 03/01/2022 | symptomatic (cough, sore throat) and contact tracing | 01/01/2022 | DaAN Gene | 24.9 (Positive) | 26.5 (Positive) |
| 042 | 12 | F | 03/01/2022 | symptomatic (cough, headache) | 01/01/2022 | DaAN Gene | 16.0 (Positive) | 26.6 (Positive) |
| 043 | 11 | M | 04/01/2022 | symptomatic (cough, fever, sore throat) | 29/12/2021 | DaAN Gene | 22.4 (Positive) | 29.1 (Positive) |
| 044 | 11 | F | 04/01/2022 | symptomatic (headache) and contact tracing | 03/01/2022 | DaAN Gene | Negative | Negative |
| 045 | 11 | M | 04/01/2022 | symptomatic (cough) and contact tracing | 03/01/2022 | DaAN Gene | 22.4 (Positive) | 26.1 (Positive) |
| 046 | 16 | F | 04/01/2022 | symptomatic (blocked nose, sore throat) and contact tracing | 31/12/2021 | DaAN Gene | Negative | Negative |
| 047 | 9 | F | 04/01/2022 | contact tracing, no symptoms | n/a | DaAN Gene | Negative | Negative |
| 048 | 7 | F | 04/01/2022 | contact tracing, no symptoms | n/a | DaAN Gene | Negative | Negative |
| 049 | 15 | M | 04/01/2022 | contact tracing, no symptoms | n/a | DaAN Gene | Negative | Negative |
| 50 | 12 | M | 04/01/2022 | symptomatic (cough, headache, sore throat) | 01/01/2022 | DaAN Gene | 20.3 (Positive) | 33.0 (Positive) |
| 051 | 12 | M | 04/01/2022 | symptomatic (sore throat) | 02/01/2022 | DaAN Gene | 19.2 (Positive) | 33.1 (Positive) |
| 052 | 12 | M | 05/01/2022 | symptomatic (cough, sneezing) | 03/01/2022 | DaAN Gene | 23.6 (Positive) | 28.0 (Positive) |
| 053 | 5 | M | 05/01/2022 | contact tracing, no symptoms | n/a | DaAN Gene | Negative | Negative |
| 054 | 41 | F | 05/01/2022 | symptomatic (dry throat) and contact tracing | 02/01/2022 | DaAN Gene | Negative | Negative |
| 055 | 7 | M | 05/01/2022 | contact tracing, no symptoms | n/a | DaAN Gene | 34.2 (Positive) | Negative |
| 056 | 34 | F | 05/01/2022 | contact tracing, no symptoms | n/a | DaAN Gene | 37.9 (Negative) | 39.5 (Negative) |
| 057 | 12 | M | 05/01/2022 | symptomatic (cough, headache, sore throat) | 02/01/2022 | DaAN Gene | Negative | Negative |
| 058 | 15 | M | 05/01/2022 | contact tracing, no symptoms | n/a | DaAN Gene | Negative | Negative |
| 059 | 59 | F | 05/01/2022 | symptomatic (apnoea, diarrhea, headache, chest pains) | 02/01/2022 | DaAN Gene | Negative | Negative |
| 060 | 11 | F | 05/01/2022 | Second test | n/a | DaAN Gene | Negative | Negative |
| 061 | 9 | F | 05/01/2022 | Second test | n/a | DaAN Gene | Negative | Negative |
| 062 | 44 | F | 05/01/2022 | Second test | n/a | DaAN Gene | Negative | Negative |
| 063 | 14 | M | 06/01/2022 | symptomatic (cough, sneezing, blocked nose) | 02/01/2022 | DaAN Gene | 16.6 (Positive) | 31.3 (Positive) |
| 064 | 38 | F | 06/01/2022 | symptomatic (sore throat) | 06/01/2022 | DaAN Gene | Negative | Negative |
| 065 | 32 | F | 06/01/2022 | symptomatic (cough, sneezing, sore throat) | 04/01/2022 | DaAN Gene | 37.5 (Negative) | 32.7 (Positive) |
| 066 | 9 | F | 06/01/2022 | symptomatic (sneezing) | 03/01/2022 | DaAN Gene | 36.2 (Negative) | Negative |
| 067 | 21 | M | 06/01/2022 | symptomatic (sore throat) and contact tracing | 04/01/2022 | DaAN Gene | Negative | Negative |
| 068 | 15 | M | 06/01/2022 | symptomatic (headache, fever, dizziness, sore throat) and contact tracing | 04/01/2022 | DaAN Gene | 14.6 (Positive) | 27.6 (Positive) |
| 069 | 8 | M | 06/01/2022 | symptomatic (running nose, sneezing) and contact tracing | 06/01/2022 | DaAN Gene | Negative | Negative |
| 070 | 18 | F | 06/01/2022 | symptomatic (running nose) and contact tracing | 01/01/2022 | DaAN Gene | Negative | Negative |
| 071 | 45 | F | 06/01/2022 | symptomatic (headache, sneezing) | 05/01/2022 | DaAN Gene | Negative | Negative |
| 072 | 12 | M | 06/01/2022 | no symptoms | n/a | DaAN Gene | Negative | Negative |
| 073 | 9 | F | 11/01/2022 | symptomatic (cough) and contact tracing | 09/01/2022 | DaAN Gene | 33.6 (Positive) | Negative |
| 074 | 45 | M | 11/01/2022 | symptomatic (cough) and contact tracing | 09/01/2022 | DaAN Gene | 18.0 (Positive) | 28.5 (Positive) |
| 075 | 6 | M | 11/01/2022 | contact tracing, no symptoms | n/a | DaAN Gene | Negative | Negative |
| 076 | 16 | F | 12/01/2022 | symptomatic (sore throat) | 10/01/2022 | DaAN Gene | Negative | Negative |
| 077 | 14 | F | 12/01/2022 | contact tracing, no symptoms | n/a | DaAN Gene | Negative | Negative |
| 078 | 17 | M | 12/01/2022 | contact tracing, no symptoms | n/a | DaAN Gene | Negative | Negative |
| 079 | 11 | M | 12/01/2022 | symptomatic (cough, headache) and contact tracing | 05/01/2022 | DaAN Gene | 26.7 (Negative) | 40.7 (Negative) |
| 080 | 6 | F | 13/01/2022 | contact tracing, no symptoms | n/a | DaAN Gene | Negative | Negative |
| 081 | 48 | F | 13/01/2022 | contact tracing, no symptoms | n/a | DaAN Gene | 41.8 (Negative) | Negative |
| 082 | 5 | F | 14/01/2022 | symptomatic (cough) and contact tracing | 11/01/2022 | DaAN Gene | 39.9 (Negative) | Negative |
| 083 | 11 | F | 18/01/2022 | symptomatic (cough) | 11/01/2022 | DaAN Gene | Negative | Negative |
| 084 | 12 | F | 19/01/2022 | symptomatic (cough) | 17/01/2022 | DaAN Gene | Negative | Negative |
| 085 | 5 | F | 19/01/2022 | symptomatic (sneezing) and contact tracing | 17/01/2022 | DaAN Gene | Negative | Negative |
| 086 | 43 | F | 19/01/2022 | contact tracing, no symptoms | n/a | DaAN Gene | 22.9 (Positive) | 26.3 (Positive) |
| 087 | 11 | F | 19/01/2022 | symptomatic (blocked nose) and contact tracing | 18/01/2022 | DaAN Gene | 15.8 (Positive) | 31.5 (Positive) |
| 088 | 46 | M | 19/01/2022 | contact tracing, no symptoms | n/a | DaAN Gene | 22.4 (Positive) | 21.5 (Positive) |
| 089 | 11 | M | 19/01/2022 | symptomatic (cough) | 17/01/2022 | DaAN Gene | 34.3 (Positive) | Negative |
| 090 | (ADULT) | M | 19/01/2022 | Routine checkup, no symptoms | n/a | DaAN Gene | Negative | Negative |
| 091 | 16 | M | 19/01/2022 | contact tracing, no symptoms | n/a | DaAN Gene | 30.7 (Positive) | 36.1 (Negative) |
| 092 | 12 | M | 19/01/2022 | symptomatic (sneezing) and contact tracing | 18/01/2022 | DaAN Gene | Negative | Negative |
| 093 | 31 | F | 27/07/2022 | symptomatic (blocked nose, cough, headache, sore throat) | 25/07/2022 | GeneXpert SARS-CoV-2 | Negative | Negative |
| 094 | 39 | F | 27/07/2022 | contact tracing, no symptoms | n/a | GeneXpert SARS-CoV-2 | Negative | Negative |
| 095 | 28 | F | 27/07/2022 | symptomatic (cough, fatigue, headache, shivering, stiff neck) | 25/07/2022 | GeneXpert SARS-CoV-2 | Negative | Negative |
| 096 | 24 | M | 27/07/2022 | symptomatic (fever, headache, sore throat) | 25/07/2022 | GeneXpert SARS-CoV-2 | Negative | Negative |
| 097 | 51 | M | 28/07/2022 | symptomatic (blocked nose, cough, fever, shortness of breath, sore throat) | 25/07/2022 | GeneXpert SARS-CoV-2 | Negative | Negative |
| 098 | 18 | F | 28/07/2022 | symptomatic (cough, fever, headache, sore throat, vomiting) | 07/07/2022 | GeneXpert SARS-CoV-2 | Negative | Negative |
| 099 | 48 | M | 01/08/2022 | symptomatic (blocked nose, cough, headache) | 29/07/2022 | GeneXpert SARS-CoV-2 | Negative | Negative |
| 100 | 32 | F | 02/08/2022 | symptomatic (dizziness, headache) | 30/07/2022 | GeneXpert SARS-CoV-2 | Negative | Negative |
| 101 | 23 | F | 02/08/2022 | symptomatic (cough, headache) | 31/07/2022 | GeneXpert SARS-CoV-2 | Negative | Negative |
| 102 | 43 | F | 02/08/2022 | symptomatic (headache, sore throat) | 01/08/2022 | GeneXpert SARS-CoV-2 | Negative | Negative |
| 103 | 39 | M | 03/08/2022 | symptomatic (headache) and contact tracing | 01/08/2022 | GeneXpert SARS-CoV-2 | Negative | Negative |
| 104 | 64 | M | 03/08/2022 | symptomatic (headache) and contact tracing | 31/07/2022 | GeneXpert SARS-CoV-2 | Negative | Negative |
| 105 | 25 | F | 04/08/2022 | symptomatic (abdominal pain, diarrhea, fever) | 02/08/2022 | GeneXpert SARS-CoV-2 | Negative | Negative |
| 106 | 18 | F | 10/08/2022 | symptomatic (headache, sneezing) | 06/08/2022 | GeneXpert SARS-CoV-2 | Negative | Negative |
| 107 | 41 | F | 10/08/2022 | symptomatic (body ache, chills, cough, fever, headache, night sweats) | 06/08/2022 | GeneXpert SARS-CoV-2 | Negative | Negative |
| 108 | 34 | F | 10/08/2022 | symptomatic (chest pain, sneezing) | 01/08/2022 | GeneXpert SARS-CoV-2 | Negative | Negative |
| 109 | 35 | F | 10/08/2022 | symptomatic (blocked nose, cough, fatigue, fever, headache, sore throat) | 09/08/2022 | GeneXpert SARS-CoV-2 | Negative | Negative |
| 110 | 21 | M | 11/08/2022 | symptomatic (cough, headache, sore throat) | unknown | GeneXpert SARS-CoV-2 | Negative | Negative |
| 111 | 57 | F | 15/08/2022 | symptomatic (cough, nasal congestion, sore throat) | 08/08/2022 | GeneXpert SARS-CoV-2 | Negative | Negative |
| 112 | 37 | F | 15/08/2022 | symptomatic (cough, fever, sore throat) | 09/08/2022 | GeneXpert SARS-CoV-2 | Negative | Negative |
| 113 | 34 | M | 15/08/2022 | symptomatic (fever, headache, loss of taste, shortness of breath, sore threat) | 14/08/2022 | GeneXpert SARS-CoV-2 | Negative | Negative |
| 114 | 30 | F | 16/08/2022 | symptomatic (fever, headache, nasal congestion, sore throat) | 15/08/2022 | GeneXpert SARS-CoV-2 | Negative | Negative |
| 115 | 34 | M | 16/08/2022 | symptomatic (fatigue, fever, headache, sore throat) | 15/08/2022 | GeneXpert SARS-CoV-2 | Negative | Negative |
| 116 | 43 | M | 18/08/2022 | symptomatic (cough, headache, sore throat) | 16/08/2022 | GeneXpert SARS-CoV-2 | Negative | Negative |
| 117 | 45 | M | 24/08/2022 | symptomatic (cough, red eyes, sore throat) | 22/08/2022 | GeneXpert SARS-CoV-2 | Negative | Negative |
| 118 | 34 | F | 25/08/2022 | symptomatic (congestion, cough, headache, stiff neck, sore throat) | 22/08/2022 | GeneXpert SARS-CoV-2 | Negative | Negative |
| 119 | 25 | M | 28/08/2022 | symptomatic (cough, running nose, sore throat) | 27/08/2022 | GeneXpert SARS-CoV-2 | Negative | Negative |
| 120 | 22 | F | 29/08/2022 | symptomatic (chest pain, cough, diarrhea, headache, running nose, trembling) | 28/08/2022 | GeneXpert SARS-CoV-2 | Negative | Negative |
| 121 | 24 | F | 05/09/2022 | Routine checkup, no reported symptoms | n/a | GeneXpert SARS-CoV-2 | Negative | Negative |
| 122 | 28 | M | 07/09/2022 | symptomatic (dry cough, fatigue, loss of appetite) | 06/09/2022 | GeneXpert SARS-CoV-2 | Negative | Negative |
| 123 | 36 | M | 08/09/2022 | symptomatic (backache, cough, fever, headache) | 07/09/2022 | GeneXpert SARS-CoV-2 | Negative | Negative |
| 124 | Not provided | M | 12/09/2022 | symptomatic (chest pain, diarrhea, dizziness, fever, headache, joint pains, sore throat, vomiting) | 11/09/2022 | GeneXpert SARS-CoV-2 | Negative | Negative |
| 125 | 54 | F | 14/09/2022 | symptomatic (body pain, cough, diarrhea, dizziness, headache, shortness of breath, sore throat) | 11/09/2022 | GeneXpert SARS-CoV-2 | Negative | Negative |
| 126 | 58 | M | 14/09/2022 | symptomatic (cough, running nose, sore throat) | 10/09/2022 | GeneXpert SARS-CoV-2 | Negative | Negative |
| 127 | 30 | F | 16/09/2022 | symptomatic (blocked nose, chest pains, chills, cough, fever, headache) | 13/09/2022 | GeneXpert SARS-CoV-2 | Negative | Negative |

**Supp Table 2:** Study samples subjected to NGS and phylogenetic analysis

| Sample ID | Accession Number | Genome length | Genome coverage (%) |
| --- | --- | --- | --- |
| KJL001_NOS | EPI_ISL_17960367 | 29811 | 99.8 |
| KJL001_SG | EPI_ISL_17960374 | 29620 | 88.7 |
| KJL003_NOS | EPI_ISL_17960364 | 29751 | 99.6 |
| KJL003_SG | EPI_ISL_17960371 | 29719 | 99.5 |
| KJL005_NOS | EPI_ISL_17960362 | 29761 | 99.7 |
| KJL005_SG | EPI_ISL_17960369 | 29647 | 98.4 |
| KJL009_NOS | EPI_ISL_15158767 | 29714 | 99.2 |
| KJL009_SG | EPI_ISL_15158768 | 29714 | 99.2 |
| KJL011_NOS | EPI_ISL_15158765 | 29714 | 90.0 |
| KJL011_SG | EPI_ISL_15158766 | 29714 | 91.2 |
| KJL013_NOS | EPI_ISL_17960378 | 29742 | 99.6 |
| KJL013_SG | EPI_ISL_17960381 | 28584 | 62.9 |
| KJL015_NOS | EPI_ISL_15158770 | 29714 | 99.2 |
| KJL015_SG | EPI_ISL_15158771 | 29714 | 94.7 |
| KJL019_NOS | EPI_ISL_17960361 | 29759 | 99.6 |
| KJL019_SG | EPI_ISL_17960375 | 29690 | 95.4 |
| KJL021_NOS | EPI_ISL_15158773 | 29714 | 99.2 |
| KJL021_SG | EPI_ISL_15158774 | 29714 | 99.2 |
| KJL023_NOS | EPI_ISL_17960373 | 29776 | 98.9 |
| KJL023_SG | EPI_ISL_15158775 | 29714 | 99.2 |
| KJL025_NOS | EPI_ISL_17960365 | 29760 | 99.7 |
| KJL025_SG | EPI_ISL_15158772 | 29714 | 87.1 |
| KJL027_NOS | EPI_ISL_17960359 | 29760 | 99.7 |
| KJL027_SG | EPI_ISL_17960383 | 28879 | 52.2 |
| KJL029_NOS | EPI_ISL_15158778 | 29714 | 99.2 |
| KJL029_SG | EPI_ISL_15158779 | 29714 | 99.2 |
| KJL031_NOS | EPI_ISL_17960377 | 29777 | 99.8 |
| KJL031_SG | EPI_ISL_15158777 | 29714 | 83.3 |
| KJL033_NOS | EPI_ISL_17960379 | 29638 | 98.5 |
| KJL033_SG | EPI_ISL_17960382 | 29620 | 66.4 |

**Supp Table 3:** Study samples subjected to NGS and their identified single-nucleotide polymorphisms (SNPs) on the Spike protein

| Sample ID | Spike Mutations |
| --- | --- |
| KJL001_NOS | A67V,H69del,V70del,T95I,G142del,V143del,Y144del,Y145D,N211I,L212del,G339D,S371L,S373P,S375F,K417N,N440K,G446S,S477N,T478K,E484A,Q493R,G496S,Q498R,N501Y,Y505H,T547K,D614G,H655Y,N679K,P681H,N764K,D796Y,N856K,Q954H,N969K,L981F |
| KJL001_SG | A67V,H69del,V70del,T95I,G142del,V143del,Y144del,Y145D,N211I,L212del,G339D,S371L,S373P,S375F,K417N,N440K,G446S,S477N,T478K,E484A,Q493R,G496S,Q498R,N501Y,Y505H,T547K,D614G,H655Y,N679K,P681H,D796Y,N856K,Q954H,N969K,L981F |
| KJL003_NOS | A67V,H69del,V70del,T95I,G142del,V143del,Y144del,Y145D,N211I,L212del,G339D,S371L,S373P,S375F,K417N,N440K,G446S,S477N,T478K,E484A,Q493R,G496S,Q498R,N501Y,Y505H,T547K,D614G,H655Y,N679K,P681H,N764K,D796Y,N856K,Q954H,N969K,L981F |
| KJL003_SG | A67V,H69del,V70del,T95I,G142del,V143del,Y144del,Y145D,N211I,L212del,G339D,S371L,S373P,S375F,K417N,N440K,G446S,S477N,T478K,E484A,Q493R,G496S,Q498R,N501Y,Y505H,T547K,D614G,H655Y,N679K,P681H,N764K,D796Y,N856K,Q954H,N969K,L981F |
| KJL005_NOS | A67V,H69del,V70del,T95I,G142del,V143del,Y144del,Y145D,N211I,L212del,G339D,S371L,S373P,S375F,K417N,N440K,G446S,S477N,T478K,E484A,Q493R,G496S,Q498R,N501Y,Y505H,T547K,D614G,H655Y,N679K,P681H,N764K,D796Y,N856K,Q954H,N969K,L981F |
| KJL005_SG | A67V,H69del,V70del,T95I,G142del,V143del,Y144del,Y145D,N211I,L212del,G339D,S371L,S373P,S375F,K417N,N440K,G446S,S477N,T478K,E484A,Q493R,G496S,Q498R,N501Y,Y505H,T547K,D614G,H655Y,N679K,P681H,N764K,D796Y,N856K,Q954H,N969K,L981F |
| KJL009_NOS | A67V,T95I,G339D,S371L,S373P,S375F,K417N,N440K,G446S,S477N,T478K,E484A,Q493R,G496S,Q498R,N501Y,Y505H,T547K,D614G,H655Y,N679K,P681H,N764K,D796Y,N856K,Q954H,N969K,L981F |
| KJL009_SG | A67V,T95I,G339D,S371L,S373P,S375F,K417N,N440K,G446S,S477N,T478K,E484A,Q493R,G496S,Q498R,N501Y,Y505H,T547K,D614G,H655Y,N679K,P681H,N764K,D796Y,N856K,Q954H,N969K,L981F |
| KJL011_NOS | T19I,G142D,V213G,G339D,S371F,S373P,S375F,T376A,D405N,R408S,K417N,N440K,S477N,T478K,E484A,Q493R,Q498R,N501Y,Y505H,D614G,H655Y,N679K,P681H,N764K,D796Y,Q954H,N969K |
| KJL011_SG | T19I,G142D,V213G,G339D,S371F,S373P,S375F,T376A,D405N,R408S,K417N,N440K,S477N,T478K,E484A,Q493R,Q498R,N501Y,Y505H,D614G,H655Y,N679K,P681H,N764K,D796Y,Q954H,N969K |
| KJL013_NOS | T19I,L24S,P25del,P26del,A27del,G142D,V213G,G339D,S371F,S373P,S375F,T376A,D405N,R408S,K417N,N440K,S477N,T478K,E484A,Q493R,Q498R,N501Y,Y505H,D614G,H655Y,N679K,P681H,N764K,D796Y,Q954H,N969K |
| KJL013_SG | T19I,L24S,P25del,P26del,A27del,G142D,V213G,G339D,S371F,S373P,S375F,T376A,D405N,R408S,K417N,N440K,S477N,T478K,E484A,Q493R,Q498R,N501Y,Y505H,D614G,H655Y |
| KJL015_NOS | A67V,T95I,G339D,S371L,S373P,S375F,K417N,N440K,G446S,S477N,T478K,E484A,Q493R,G496S,Q498R,N501Y,Y505H,T547K,D614G,H655Y,N679K,P681H,N764K,D796Y,N856K,Q954H,N969K,L981F |
| KJL015_SG | A67V,T95I,G339D,S371L,S373P,S375F,K417N,N440K,G446S,S477N,T478K,E484A,Q493R,G496S,Q498R,N501Y,Y505H,T547K,D614G,H655Y,N679K,P681H |
| KJL019_NOS | A67V,H69del,V70del,T95I,G142del,V143del,Y144del,Y145D,N211I,L212del,G339D,S371L,S373P,S375F,K417N,N440K,G446S,S477N,T478K,E484A,Q493R,G496S,Q498R,N501Y,Y505H,T547K,D614G,H655Y,N679K,P681H,N764K,D796Y,N856K,Q954H,N969K,L981F,D1139H |
| KJL019_SG | A67V,H69del,V70del,T95I,G142del,V143del,Y144del,Y145D,N211I,L212del,G339D,S371L,S373P,S375F,K417N,N440K,G446S,S477N,T478K,E484A,Q493R,G496S,Q498R,N501Y,Y505H,T547K,D614G,H655Y,N679K,P681H,N764K,D796Y,N856K,Q954H,N969K,L981F,D1139H |
| KJL021_NOS | A67V,T95I,G339D,R346K,S371L,S373P,S375F,K417N,N440K,G446S,S477N,T478K,E484A,Q493R,G496S,Q498R,N501Y,Y505H,T547K,D614G,H655Y,N679K,P681H,N764K,D796Y,N856K,Q954H,N969K,L981F |
| KJL021_SG | A67V,T95I,G339D,R346K,S371L,S373P,S375F,K417N,N440K,G446S,S477N,T478K,E484A,Q493R,G496S,Q498R,N501Y,Y505H,T547K,D614G,H655Y,N679K,P681H,N764K,D796Y,N856K,Q954H,N969K,L981F |
| KJL023_NOS | A67V,H69del,V70del,T95I,G142del,V143del,Y144del,Y145D,N211I,L212del,G339D,R346K,S371L,S373P,S375F,K417N,N440K,G446S,S477N,T478K,E484A,Q493R,G496S,Q498R,N501Y,Y505H,T547K,D614G,H655Y,N679K,P681H,N764K,D796Y,N856K,Q954H,N969K,L981F |
| KJL023_SG | A67V,T95I,G339D,R346K,S371L,S373P,S375F,K417N,N440K,G446S,S477N,T478K,E484A,Q493R,G496S,Q498R,N501Y,Y505H,T547K,D614G,H655Y,N679K,P681H,N764K,D796Y,N856K,Q954H,N969K,L981F |
| KJL025_NOS | A67V,H69del,V70del,T95I,G142del,V143del,Y144del,Y145D,N211I,L212del,G339D,S371L,S373P,S375F,K417N,N440K,G446S,S477N,T478K,E484A,Q493R,G496S,Q498R,N501Y,Y505H,T547K,D614G,H655Y,N679K,P681H,N764K,D796Y,N856K,Q954H,N969K,L981F |
| KJL025_SG | A67V,T95I,G339D,S371L,S373P,S375F,K417N,Q493R,G496S,Q498R,N501Y,Y505H,H655Y |
| KJL027_NOS | A67V,H69del,V70del,T95I,G142del,V143del,Y144del,Y145D,N211I,L212del,G339D,S371L,S373P,S375F,K417N,N440K,G446S,S477N,T478K,E484A,Q493R,G496S,Q498R,N501Y,Y505H,T547K,D614G,H655Y,N679K,P681H,N764K,D796Y,N856K,Q954H,N969K,L981F |
| KJL027_SG | None |
| KJL029_NOS | A67V,T95I,G339D,S371L,S373P,S375F,K417N,N440K,G446S,S477N,T478K,E484A,Q493R,G496S,Q498R,N501Y,Y505H,T547K,D614G,H655Y,N679K,P681H,N764K,D796Y,N856K,Q954H,N969K,L981F |
| KJL029_SG | A67V,T95I,G339D,S371L,S373P,S375F,K417N,N440K,G446S,S477N,T478K,E484A,Q493R,G496S,Q498R,N501Y,Y505H,T547K,D614G,H655Y,N679K,P681H,N764K,D796Y,N856K,Q954H,N969K,L981F |
| KJL031_NOS | T19I,L24S,P25del,P26del,A27del,G142D,V213G,G339D,S371F,S373P,S375F,T376A,D405N,R408S,K417N,N440K,S477N,T478K,E484A,Q493R,Q498R,N501Y,Y505H,D614G,H655Y,N679K,P681H,N764K,D796Y,Q954H,N969K |
| KJL031_SG | T19I,G142D,V213G,G339D,S371F,S373P,S375F,T376A,D405N,R408S,K417N,N440K,S477N,T478K,E484A,Q493R,Q498R,N501Y,Y505H,D614G,H655Y,N679K,P681H,N764K,D796Y |
| KJL033_NOS | T19I,L24S,P25del,P26del,A27del,G142D,V213G,G339D,S371F,S373P,S375F,T376A,D405N,R408S,K417N,N440K,S477N,T478K,E484A,Q493R,Q498R,N501Y,Y505H,D614G,H655Y,N679K,P681H,N764K,D796Y,Q954H,N969K |
| KJL033_SG | T19I,L24S,P25del,P26del,A27del,G142D,V213G,G339D,Q493R,Q498R,N501Y,Y505H,N969K |
