# Supplementary material for: Saline gargle collection method is comparable to nasopharyngeal/oropharyngeal swabbing for the molecular detection and sequencing of SARS-CoV-2 in Botswana
Source: Microbiol Spectr. 2025 May 22;13(7):e02023-24. doi: 10.1128/spectrum.02023-24 (PMC12211010; doi:10.1128/spectrum.02023-24)
Supplement: Additional experimental details — GISAID EPI_ISL IDs and the DOI for samples used to generate the phylogenetic tree. [file spectrum.02023-24-s0002.pdf]

SUPPLEMENTAL TABLE

Data Availability

GISAID Identifier: EPI_SET_250320pb
DOI: https://doi.org/10.55876/gis8.250320pb

All genome sequences and associated metadata in this dataset are published in GISAID's EpiCoV database. To view the contributors of each individual sequence with details such as accession number, Virus name, Collection date, Originating Lab and Submitting Lab and the list of Authors, visit 10.55876/gis8.250107tk

Data Snapshot

EPI_SET_250320pb is composed of 59 individual genome sequences.
The collection dates range from 2020-10-15 to 2022-01-19;
Data were collected in 1 countries and territories.
